# Gender differences in the association between metabolic syndrome and periodontal disease: the Hisayama Study

**DOI:** 10.1111/jcpe.12119

**Published:** 2013-07-08

**Authors:** Michiko Furuta, Yoshihiro Shimazaki, Toru Takeshita, Yukie Shibata, Sumio Akifusa, Nobuoki Eshima, Yutaka Kiyohara, Toshiharu Ninomiya, Yoichiro Hirakawa, Naoko Mukai, Masaharu Nagata, Yoshihisa Yamashita

**Affiliations:** 1 Section of Preventive and Public Health Dentistry, Division of Oral Health, Growth and Development, Kyushu University Faculty of Dental ScienceFukuoka, Japan; 2 Department of Health Management, School of Oral Health Science, Kyushu Dental CollegeKitakyushu, Japan; 3 Department of Biostatistics, Faculty of Medicine, Oita UniversityOita, Japan; 4 Department of Environmental Medicine, Graduate School of Medical Sciences, Kyushu UniversityFukuoka, Japan; 5 Department of Medicine and Clinical Science, Graduate School of Medical Sciences, Kyushu UniversityFukuoka, Japan

**Keywords:** gender difference, metabolic syndrome, periodontal disease, structural equation model

## Abstract

**Aims**: Periodontal disease and metabolic syndrome (MS) are more prevalent in males than in females. However, whether there is a gender difference in the association between these health conditions has not yet been investigated. This study examined the gender difference in this association, considering the definition of periodontal disease.

**Materials and Methods**: We recruited 1040 males and 1330 females, aged ≥40 years, with at least ten teeth from subjects of the 2007 Hisayama health examination. We performed a logistic regression analysis with various definitions of periodontal disease the dependent variable and MS as the independent variable. Following the analysis, the data were reanalysed with the structural equations model.

**Results**: The logistic regression analysis suggested a stronger association between periodontal disease and MS in females than that in males when periodontal disease was more severely defined. When we constructed the structural equations model in each gender, the model showed a good fit to the data of females, suggesting the association between periodontal disease and MS in females, but not in males.

**Conclusions**: Gender differences seem to exist in the association between periodontal disease and MS; MS might show a stronger association with periodontal disease in females than in males.

Furuta M, Shimazaki Y, Takeshita T, Shibata Y, Akifusa S, Eshima N, Kiyohara Y, Ninomiya T, Hirakawa Y, Mukai N, Nagata M, Yamashita Y. Gender differences in the association between metabolic syndrome and periodontal disease: the Hisayama Study. J Clin Periodontol 2013; 40: 743–752. doi: 10.1111/jcpe.12119.

Recent epidemiological studies have revealed a significant association between periodontal disease and metabolic syndrome (MS) (Shimazaki et al. 2007, D’Aiuto et al. 2008, Morita et al. 2009, Andriankaja et al. 2010, Benguigui et al. 2010, Timonen et al. 2010, Kwon et al. 2011, Han et al. 2012). Much attention has been paid to this association because the rapidly increasing number of MS cases is a serious public health issue in most developed countries. Presently, it is unknown whether there is any causal relationship between the two health conditions; indeed, cohort study is only one (Morita et al. 2010) and interventional studies of the causal relationship are few.

On the other hand, using the National Cholesterol Education Programme Expert Panel Adult Treatment Panel III criteria (Grundy et al. 2004), the prevalence of MS was found to be higher in males than in females (Oh et al. 2004, Rutter et al. 2004, Arai et al. 2006). Among young and middle-aged adults, females have lower blood pressures and serum levels of triglycerides, higher insulin sensitivity, and less abdominal fat accumulation than males (Fernandez-Real et al. 1998, Razzouk & Muntner 2009). However, the protection conferred on females is not life-long and the prevalence of MS increases with age, especially after 50–60 years old (Razzouk & Muntner 2009). MS is a cluster of risk factors for the development of cardiovascular disease, including abdominal obesity, hypertension, insulin resistance, dyslipidaemia with high triglycerides and low high-density lipoprotein cholesterol (Grundy et al. 2005, Alberti et al. 2009). Several studies have revealed significant gender differences in cardiovascular disease incidence, showing a male-to- female ratio of 1.7–3.1 in subjects under 74 years of age (Lerner & Kannel 1986, Jousilahti et al. 1999, Levy et al. 2002) and the gender difference in MS is presumed to be one of the causes of that in cardiovascular disease (Regitz-Zagrosek et al. 2006).

A similar tendency of a higher incidence of disease in males than females is also the case for periodontal disease. In the Third National Health and Nutrition Examination Survey (NHANES III), the male-to-female prevalence ratios in adults were 1.1 for a probing depth of ≥3 mm, 1.4 for a probing depth of ≥4 mm, and 1.7 for a probing depth of ≥5 mm (Albandar 2002). Japanese (Furuta et al. 2010), Filipino (Horning et al. 1992), Brazilian (Susin et al. 2005) and Tanzanian surveys (Mumghamba et al. 1995) also showed that periodontal disease was more prevalent in males than in females.

It is clear that there is a gender difference in the prevalence of both MS and periodontal disease; thus, the association between periodontal disease and MS may also be expected to have a gender difference. However, most studies have not addressed this and did not use stratified analysis, which would allow determination of whether the relationship is unique to one gender, or even opposite in males and females. Moreover, in these cases, the overall analysis might reduce or compensate for actual differences between genders. To our knowledge, there are only two reports (Andriankaja et al. 2010, Kwon et al. 2011) of the relationship between MS and periodontal disease using stratified analyses. The results of these studies are contradictory; one showed a gender difference while the other did not. One reason for this discrepancy may be the different definition of periodontal disease. Thus, the purpose of this study was to explore the existence of a gender difference in the relationship between periodontal disease and MS in Japanese adults. We hypothesized that gender difference in this relationship might be affected by the definition of periodontal disease. Both multistep of dichotomous definition and continuous definition of periodontal disease were utilized to be clarified the above hypothesis in this study.

## Materials and Methods

### Study population

This study was undertaken in 2007 in the town of Hisayama, which is a suburb of the Fukuoka metropolitan area in western Japan. The study population comprised 2861 participants aged 40–79 years (75.1% of all residents in that age group), who provided written consent to participate in the study, and underwent a comprehensive examination. Dental and medical examinations were performed on 2669 subjects, including edentulous individuals. In total, 299 subjects who had missing data or fewer than ten teeth were excluded, because it is difficult to assess their current periodontal health properly (Saito et al. 2004), and fewer teeth increase the variance of pocket depth (e.g. as the subjects have few teeth, the mean pocket depth deepens greatly on the number of teeth). Finally, 2370 subjects (1040 males, 1330 females) were used in this study.

The ethics committee of Kyushu University Faculty of Dental Science approved the study design, data collection methods and procedure for obtaining informed consent.

### Oral examination

Periodontal status was assessed using pocket depth (PD) and clinical attachment loss (AL) on the mesio-buccal and mid-buccal sites for all teeth, except for third molars since these teeth when partially impacted frequently have pseudo-pockets, based on the NHANES III method. Because bleeding on probing (BOP) is a sensitive indicator of inflammation (Greenstein 1984), the percentage of teeth that bled on probing (%BOP) was also determined as an indicator for periodontal disease. Details of the periodontal examination have been described elsewhere (Shimazaki et al. 2011). Because the relationship with MS was similar in PD and clinical AL, but slightly stronger in PD than in clinical AL (Shimazaki et al. 2007), and there was a strong correlation between PD and clinical AL (Spearman correlation coefficient *r* = 0.85 for males and *r* = 0.87 for females), in this study, we used PD and %BOP as periodontal parameters to examine the association with MS.

### General examination

Blood pressure was measured three consecutive times, after participants rested for at least 5 min., using a standard mercury sphygmomanometer, with the participants in the sitting position, and the average value was used for the analysis. A blood sample was collected from the antecubital vein in the morning after an overnight fast, and the serum cholesterol, triglycerides, and fasting plasma glucose levels were measured according to methods described previously (Kubo et al. 1999). Trained nurses measured the participants’ waist circumferences at the level of the umbilicus. MS was defined based on Joint Interim Societies (Alberti et al. 2009) and elevated waist circumference determination was based on the International Obesity Task Force central obesity criteria for Asians (World Health Organization 2000), according to the International Diabetes Federation’s suggestion that ethnic-specific cut-off values of waist circumference are appropriate for diagnosing MS (Grundy et al. 2005): elevated waist circumference (≥90 cm in males, and ≥80 cm in females), elevated triglycerides (≥150 mg/dl or drug treatment for elevated triglycerides), reduced HDL (<40 mg/dl in males and <50 mg/dl in females, or drug treatment for reduced HDL), elevated blood pressure (systolic blood pressure ≥130 mmHg or diastolic blood pressure ≥85 mmHg, or anti-hypertensive drug treatment) and elevated fasting glucose (≥100 mg/dl or drug treatment for elevated glucose).

### Questionnaire

Information on smoking habits, alcohol intake, medication use and toothbrushing frequency was obtained using a self-administered questionnaire. Smoking habit was expressed as the Brinkman Index (number of cigarettes consumed per day multiplied by years of smoking) (Brinkman & Coates 1963). Alcohol intake was assessed on the basis of information about the usual weekly intake of alcoholic beverages, such as sake (rice wine), shochu (distilled liquor), beer or whisky. Moreover, alcohol intake was converted into a daily equivalent in terms of the number of *go*, a traditional Japanese unit of volume for *sake* (1 *go* = 0.18 l and contains 23 g of ethanol). In addition, subjects were asked about medication use, such anti-hypertensive, anti-hyperlipidemic and anti-diabetes drugs.

### Statistical analysis

Although PD and MS components are continuous variables, to compare previous studies, the association between two variables was checked by categorized them. The chi-square test with significant level 0.05 was used for testing the differences in these variables between males and females. To illustrate the association between periodontal disease as dependent variable and MS components as independent variable when categorizing them in logistic regression analysis, the following criteria for periodontal disease were employed: mean PD cut-off values of ≥2.0, 2.5, 3.0 and 3.5 mm. In addition, we evaluated the interaction between gender and MS components with adjustment for age, smoking habits, alcohol intake, toothbrushing frequency and present number of teeth. We also tested for effect modification for periodontal disease by age, smoking habits and alcohol intake in each gender. Pearson’s correlation coefficient was used to assess the correlation among continuous PD, %BOP and MS component data. Of these, variables with high skewness or kurtosis (i.e. triglycerides and fasting glucose) were transformed with a natural log function. The SPSS software (ver. 19.0 for Windows; IBM SPSS Japan, Tokyo, Japan) was used for data analyses.

To examine quantitatively the association between periodontal disease and MS, we conducted structural equation modelling (SEM), using the M-plus statistical package. On the basis of our hypothesis, we examined the mutual relationship between periodontal disease and MS in SEM. SEM is a statistical method to analyse a correlation and causal relationship among a system of observed and latent variables. Latent variables are not directly observable, but are rather inferred its existence by properties of multiple observed variables.

**Fig 1:**
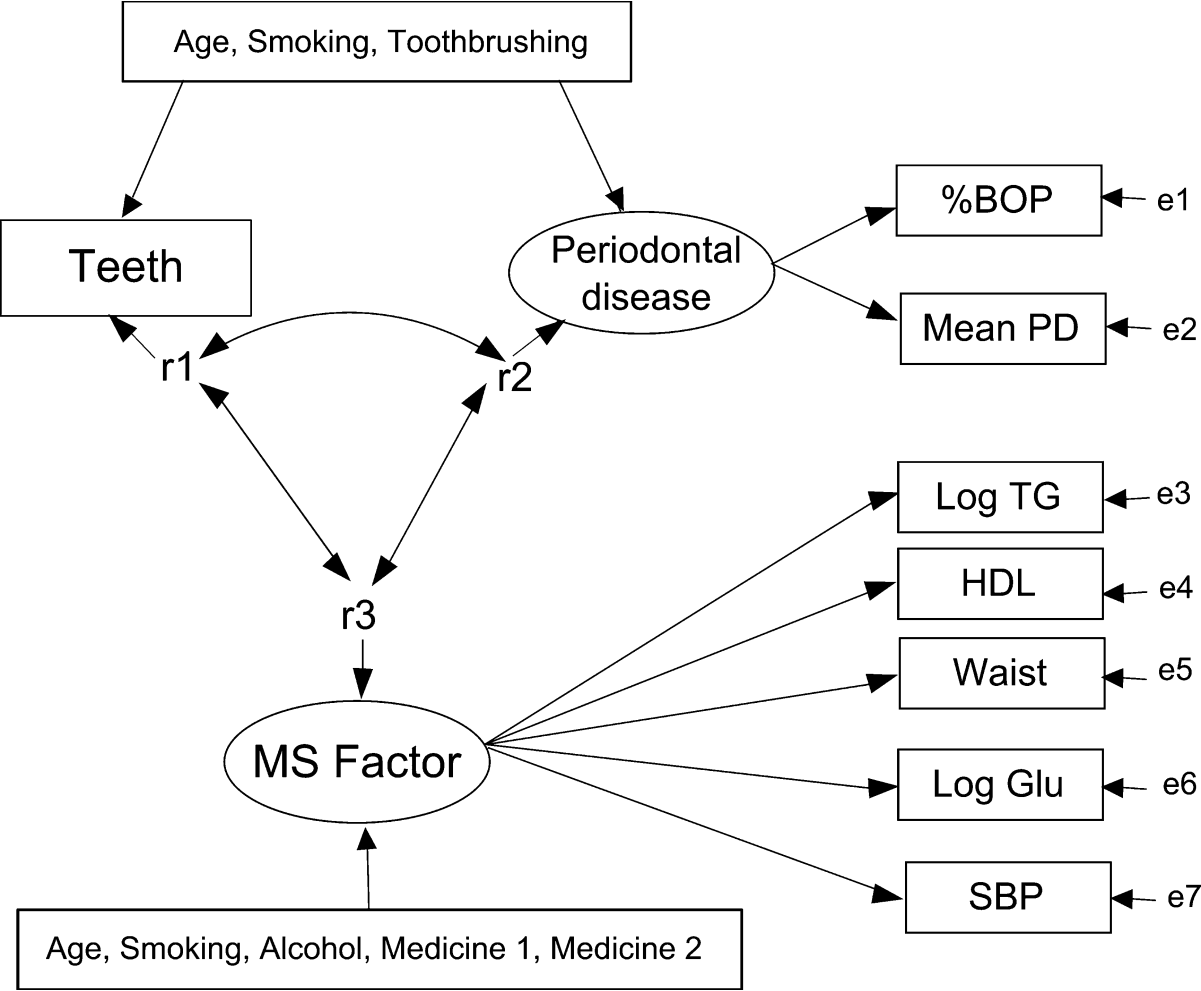
The metabolic syndrome (MS) single-factor model (Model 1). Rectangles indicate observed variables and ovals show latent constructs. Single-headed arrows indicate the directions of causal effects relating to the variables concerned, and the double-headed arrows imply the associations among the variables. The fit of Model 1 was *χ*^2^ (42) = 362.198; CFI = 0.807; RMSEA = 0.086 (0.078–0.094); SRMR = 0.055 in males and *χ*^2^ (42) = 358.831; CFI = 0.865; RMSEA = 0.075 (0.068–0.083); SRMR = 0.045 in females. The e1–e7 and r1–r3 designators refer to error terms and residual terms.

First, we defined two latent variables in periodontal disease and MS (Model 1 in Fig. 1). The MS factor was represented by all MS components, and periodontal disease consisted of PD and %BOP. We used several variables, such as age, smoking habit, alcohol intake and toothbrushing frequency, as predictors of periodontal disease, the number of teeth and MS factors. The model included error terms which referred to all unobserved sources of variance, and residual terms which represented the variance not explained by the predictors. The correlation between residual terms describes the relationship between periodontal disease factor and MS factor while omitting the effects of the variables used as the predictors.

**Fig 2:**
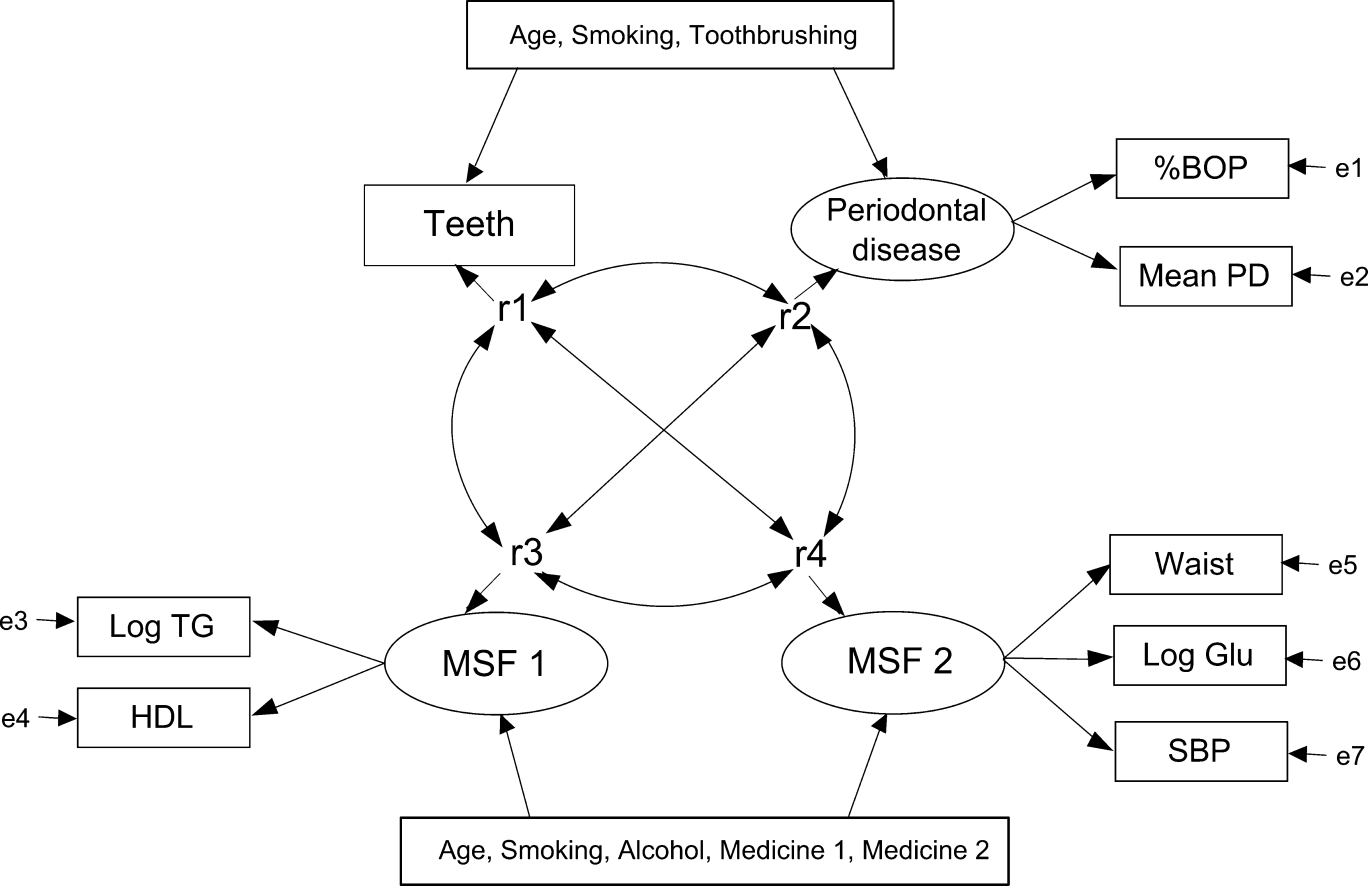
The metabolic syndrome (MS) two-factor model (Model 2). MS factor 1 (MSF1) is represented by HDL and triglyceride (log TG), and MS factor 2 (MSF2) by waist circumstance (Waist), fasting glucose (log Glu) and systolic blood pressure (SBP). Periodontal disease is represented by mean PD and %BOP. “Medicine 1” indicates anti-hyperlipidemic drug use and “Medicine 2” is anti-hypertensive drug or anti-diabetes drug use. The e1–e7 and r1–r4 designators refer to error terms and residual terms.

Next, we tested an alternative MS factor structure. Because the MS components were highly inter-related, we used statistical analysis of clustering (factor analysis). Factor analysis is a technique for reducing the number of original variables into fewer summary factors. Absolute values of factor loadings ≥0.40 after orthogonal rotation of the correlation matrix minimizing the number of variables with high loading on each factor were commonly considered for factor interpretation. According to the results of the factor analysis, two latent factors in MS components, that is, MS factors (MSF) 1 and 2, could be identified. With regard to the medications variable, we used anti-hyperlipidemic drug use as “Medicine 1” and anti-hypertensive drug or anti-diabetes drug use as “Medicine 2.” We examined correlations among residual terms (r1–r4) in MSF 1, MSF2, periodontal disease factors and the number of teeth (Fig. 2).

We used significance level 0.05 for the regression coefficients. The goodness-of-fit of the model used was assessed with a comparative fit index (CFI), a root-mean-square error of approximation (RMSEA), and the standardized root mean-square residual (SRMR). Values of close to 0.95 for the CFI, close to 0.06 for the RMSEA, and close to 0.08 for the SRMR are considered to indicate a good fit of the data to the model (Hu & Bentler 1999).

## Results

### Characteristics of subjects

Health characteristics of the subjects are summarized in Table 1. The overall mean PD was 2.5 ± 0.8 mm (mean ± SD) for males and 2.2 ± 0.7 mm for females. Females had a significantly lower mean PD (*p* < 0.001) and %BOP (*p* = 0.019) than males. There were significant differences in all MS components between males and females. There were 381 (36.6%) males with three or more of the five MS components and 451 (33.9%) females (Table 1).

**Table 1:** Demography of study subjects [*n* (%)]

Variable	Males (*n* = 1040)	Females (*n* = 1330)	All (*n* = 2370)	*p*-value
%BOP*	19.4 ± 20.9	17.5 ± 19.0	18.3 ± 19.9	0.019
Mean PD (mm)*	2.5 ± 0.8	2.2 ± 0.7	2.3 ± 0.8	<0.001
High-density lipoprotein (HDL) (mg/dL)*	61.0 ± 16.4	72.4 ± 17.7	67.4 ± 18.1	<0.001
Normal (males ≥ 40, females ≥ 50)	979 (94.1)	1221 (91.8)	2200 (92.8)	0.029
Reduced HDL (males < 40, females < 50)	61 (5.9)	109 (8.2)	170 (7.2)	
Triglycerides (mg/dl)*	154.2 ± 140.0	107.5 ± 71.0	128.0 ± 109.3	<0.001
Normal (<150)	628 (60.4)	962 (72.3)	1590 (67.1)	<0.001
Elevated triglycerides (≥150)†	412 (39.6)	368 (27.7)	780 (32.9)	
Systolic blood pressure (mmHg)*	133.2 ± 17.7	127.9 ± 19.1	130.2 ± 18.7	<0.001
Diastolic blood pressure (mmHg)*	82.3 ± 10.2	77.5 ± 10.8	79.6 ± 10.8	<0.001
Normal (<130/<85)	381 (36.6)	648 (48.7)	1029 (43.4)	<0.001
Elevated blood pressure (≥130/≥85)‡	659 (63.4)	682 (51.3)	1341 (56.6)	
Waist circumference (cm)*	86.8 ± 7.9	84.3 ± 10.2	85.4 ± 9.4	<0.001
Normal (males < 90, females < 80)	671 (64.5)	442 (33.2)	1113 (47.0)	<0.001
Elevated waist (males ≥ 90, females ≥ 80)	369 (35.5)	888 (66.8)	1257 (53.0)	
Fasting glucose (mg/dl)*	108.6 ± 24.5	101.4 ± 19.4	104.5 ± 22.1	<0.001
Normal (<100)	404 (38.8)	809 (60.8)	1213 (51.2)	<0.001
Elevated fasting glucose (≥100)§	636 (61.2)	551 (39.2)	1157 (48.8)	
Number of metabolic syndrome components				0.033
0 component	127 (12.2)	211 (15.9)	338 (14.3)	
1 or 2 components	532 (51.2)	668 (50.2)	1200 (50.6)	
≥3 components (metabolic syndrome)	381 (36.6)	451 (33.9)	832 (35.1)	
Body mass index*	23.7 ± 3.1	23.0 ± 3.6	23.3 ± 3.4	<0.001
Age	59.5 ± 10.1	59.5 ± 10.1	59.5 ± 10.1	0.972
Smoking habits (Brinkman index)	578.7 ± 511.6	44.6 ± 154.0	279.0 ± 445.4	<0.001
Alcohol intake (*go*/day)	1.5 ± 1.7	0.2 ± 0.5	0.8 ± 1.4	<0.001
Toothbrushing frequency per day				<0.001
No/1 time	484 (46.5)	271 (20.4)	755 (31.9)	
2 times	432 (41.5)	796 (59.8)	1228 (51.8)	
More than 3 times	124 (11.9)	263 (19.8)	387 (16.3)	
Number of teeth present*	24.6 ± 5.0	24.1 ± 4.8	24.3 ± 4.9	0.015

* Mean ± SD.

† Triglycerides ≥ 150 mg/dl or drug treatment for dyslipidaemia.

‡ Systolic blood pressure ≥ 130 mmHg or diastolic blood pressure ≥ 85 mmHg, or anti-hypertensive drug treatment.

§ Fasting glucose ≥ 100 mg/dl or drug treatment for elevated glucose.

### Logistic regression analysis

For the analysis technique, we need to determine the direction of the relationship in the logistic regression analysis. Therefore, we provisionally defined periodontal disease and MS as dependent and independent variables respectively. At first, we evaluated the effect of varying criteria of periodontitis (the mean PD ≥2.0, 2.5, 3.0 or 3.5 mm) on its association with MS. Setting the periodontal disease as a binary response variable, that is, 1 for positive and 0 for negative, a logistic regression analysis was performed, where explanatory variables are MS components, age, smoking habits, etc. The crude and adjusted odds ratios with respect to MS components for the above different criteria of periodontal diseases were estimated as in Table 2. In the results in females, three or more MS components were associated significantly with periodontal disease, defined by criteria mean PD ≥2.0, 2.5, 3.0 and 3.5 mm, compared with those with no MS components. In males, there was significant association between MS and periodontal disease, defined by criteria mean PD ≥2.0 and 2.5 mm. When we investigated the interaction between gender and MS components, the interaction term consisting of gender and three or more MS components was significant (*p* = 0.036) for periodontal disease defined by a mean PD ≥3.0 mm. In addition, in performing tests of interaction for periodontal disease among age, smoking habits and alcohol intake (interaction terms: age × smoking, age × alcohol, smoking × alcohol, age × smoking × alcohol), there was no statistically significant effects in each gender.

**Table 2:** Periodontal disease odds ratios according to mean PD cut-off value in males and females

Accumulation of metabolic components	Males			Females			Interaction for gender × metabolic component *p*-value†
	No. of subjects with periodontal disease	Crude OR (95% CI)		Adjusted OR* (95% CI)		No. of subjects with periodontal disease		Crude OR (95% CI)		Adjusted OR* (95% CI)	
Mean PD ≥ 2.0 mm	788 (75.6)					782 (58.8)					
Number of metabolic components											
0 component	86 (67.2)	1		1		99 (46.9)	1		1		
1 and 2 components	392 (73.5)	1.36	(0.90–2.06)	1.18	(0.77–1.82)	383 (57.2)	1.52	(1.11–2.07)	1.28	(0.93–1.78)	0.553
≥3 components (metabolic syndrome)	310 (81.4)	2.13	(1.36–3.35)	1.64	(1.02–2.64)	300 (66.5)	2.25	(1.61–3.14)	1.55	(1.07–2.24)	0.790
Mean PD ≥ 2.5 mm	408 (39.2)					334 (25.1)					
Number of metabolic components											
0 component	33 (25.8)	1		1		31 (14.7)	1		1		
1 and 2 components	198 (37.1)	1.70	(1.10–2.62)	1.41	(0.90–2.22)	155 (23.2)	1.75	(1.15–2.67)	1.45	(0.93–2.25)	0.700
≥3 components (metabolic syndrome)	177 (43.3)	2.50	(1.60–3.90)	1.86	(1.17–2.97)	148 (32.8)	2.84	(1.85–4.36)	1.87	(1.17–2.98)	0.640
Mean PD ≥ 3.0 mm	225 (21.6)					137 (10.3)					
Number of metabolic components											
0 component	19 (14.8)	1		1		9 (4.3)	1		1		
1 and 2 components	117 (22.0)	1.60	(0.94–2.72)	1.36	(0.78–2.35)	59 (8.8)	2.17	(1.06–4.46)	2.00	(0.95–4.21)	0.303
≥3 components (metabolic syndrome)	89 (23.4)	1.73	(1.00–2.98)	1.32	(0.75–2.34)	69 (15.3)	4.05	(1.98–8.29)	3.06	(1.42–6.59)	0.036
Mean PD ≥ 3.5 mm	116 (11.1)					63 (4.7)					
Number of metabolic components											
0 component	11 (8.6)	1		1		3 (1.4)	1		1		
1 and 2 components	56 (10.5)	1.25	(0.63–2.46)	1.03	(0.51–2.07)	28 (4.2)	3.03	(0.91–10.06)	2.59	(0.76–8.86)	0.133
≥3 components (metabolic syndrome)	49 (12.9)	1.57	(0.79–3.12)	1.21	(0.59–2.49)	32 (7.1)	5.30	(1.60–17.49)	3.60	(1.03–12.61)	0.066

Logistic regression analysis with periodontal disease (mean PD cut-off value ≥2.0, 2.5, 3.0, 3.5 or 4.0 mm) as the dependent variable and accumulation of metabolic components as the independent variable.

* Adjusted for age, smoking habits, alcohol intake, toothbrushing frequency and present number of teeth.

† *p*-value of the interaction term consisting of gender and MS components were calculated by using gender, MS components, age, smoking habits, alcohol intake, toothbrushing frequency and present number of teeth as explanatory variables. The interaction term was created by multiplying gender variable (0 = male, 1 = female) by MS components variable (0 = 0 component, 1 = 1 and 2 component, 2 = 3 or more components).

In this study, the percentage of subjects with elevated fasting glucose (i.e. fasting glucose ≥100 mg/dl or drug treatment for elevated glucose) were significantly higher in males than in females (61.2% males, 39.2% females). Since males were more likely to have drug treatment than females (9.0% males, 5.6% females), it was possible that drug treatment affected elevated fasting glucose as one of MS components. Therefore, we evaluated the effect of MS components on periodontal disease, while adjusting for drug treatment, age, smoking habits, etc., in Table S2. The result was similar to that previously adjusted for drug treatment.

We found gender difference in the association between MS and periodontal disease defined by a mean PD ≥3.0 or 3.5 mm, but not for periodontal disease defined by a mean PD ≥2.0 or 2.5 mm suggesting that the result from the logistic regression analysis depends on different categorical definition of periodontal disease (i.e. different mean PD cut-off values). Therefore, it is desirable to treat PD as a continuous variable and we utilized SEM, an analytical technique, for continuous variables to assess the association between periodontal disease and MS qualitatively.

### Factor analysis of MS components and structural equation modelling

To examine the association between periodontal disease and MS, the correlation coefficients among the manifest variables for assessing them were estimated (Table S1). While all of the MS components were statistically correlated with PD and %BOP in females at the level of significant 0.05, some MS components were not in males. Systolic blood pressure was used as blood pressure in further analyses due to the strong correlation between systolic and diastolic blood pressure (Spearman correlation coefficient *r* = 0.87 for males and *r* = 0.89 for females). Although the correlation analysis was performed for the manifest variables concerning periodontal disease and MS, we regarded the states of them as latent variables, and to evaluate the correlations among the latent variables a SEM approach was carried out.

First, we considered the MS single-factor model (Model 1) shown in Fig. 1. This path system has two latent variables, that is, “MS factor” and “Periodontal disease.” Analysing the data of males and females, Model 1 did not show good fits to both of males and females data sets, that is, CFI = 0.807 for males and CFI = 0.865 for females.

Second, to construct an alternative MS factor structure, factor analysis of MS components was carried out. According to Table 3, the results in males and females are similar, and two factors, which are referred to as MSF1 and 2, were extracted from the male’s and female’s data respectively. Considering the factor loadings, MSF1 is related to HDL and log triglycerides, and MSF2 relates systolic blood pressure, waist and log fasting glucose.

**Table 3:** Factor analysis of metabolic components, factors and factor loadings

	Males		Females	
MSF1	MSF2	MSF1	MSF2
HDL	0.99	0.02	0.95	−0.10
Log triglycerides	−0.47	0.29	−0.49	0.36
Systolic blood pressure	−0.02	0.64	−0.10	0.63
Waist	−0.35	0.43	−0.11	0.52
Log fasting glucose	−0.09	0.37	−0.20	0.51
% Total variance	38.2	22.6	42.70	20.6
% Cumulative variance	38.2	60.8	42.70	63.3

MSF, MS factor.

**Fig 3:**
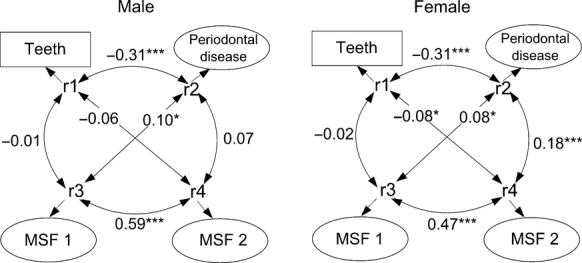
The metabolic syndrome (MS) two-factor model (Model 2) for males and females. Significant values are *p < 0.05, **p < 0.01, ***p < 0.001. The fit of Model 2 was *χ*^2^ (35) = 238.203; CFI = 0.877; RMSEA = 0.075 (0.066–0.084); SRMR = 0.042 in males and *χ*^2^ (35) = 118.504; CFI = 0.964; RMSEA = 0.042 (0.034–0.051); SRMR = 0.028 in females. The e1–e7 and r1–r4 designators refer to error terms and residual terms.

Third, according to factor analysis mentioned above, two latent variables for MS factors, that is, “MSF1” and “MSF2” were constructed in SEM (Model 2). The path diagram is shown in Fig. 2, and the estimates of the path coefficients in Model 2 are shown in Table 4. The latent variables “MSF1” and “MSF2” were represented by HDL and triglyceride, and by waist circumstance, fasting glucose and systolic blood pressure respectively. The latent variable “Periodontal disease” was represented by mean PD and %BOP. The correlations among “MSF1,” “MSF2,” “Periodontal disease” and “Teeth” in males and females are shown in Fig. 3. The correlation between residual terms indicates the relationship which was omitted the effects of several variables such as age, smoking habit, alcohol intake, medicine use and toothbrushing frequency. The effect of these variables on “MSF1,” “MSF2,” “Periodontal disease” and “Teeth” are also shown in Fig. 3. In females, Model 2 fits the data well [*χ*^2^ (35) = 118.504; CFI = 0.964; RMSEA = 0.042 (0.034–0.051); SRMR = 0.028]. In females, Model 2 showed that “Periodontal disease” correlated significantly with “MSF1” (the correlation coefficient = 0.08, *p* = 0.027), “MSF2” (the correlation coefficient = 0.18, *p* < 0.001), adjusted for several variables such as age, smoking habits. In males, Model 2 did not fit the observed data well [*χ*^2^ (35) = 238.203; CFI = 0.877; RMSEA = 0.075 (0.066–0.084); SRMR = 0.042]. The causal and correlation structure in males may be more complex than that in females.

**Table 4:** Maximum-likelihood parameter estimates for the structural regression model of periodontal disease and metabolic syndrome

Parameter	Males			Females		
	*β*	SE	*p*-value	*β*	SE	*p*-value
Factor loadings						
Periodontal disease→PD	0.941	0.046	<0.001	0.809	0.032	<0.001
Periodontal disease→%BOP	0.670	0.037	<0.001	0.744	0.030	<0.001
MSF 1→Log TG	0.787	0.064	<0.001	0.881	0.042	<0.001
MSF 1→HDL	−0.581	0.051	<0.001	−0.571	0.033	<0.001
MSF 2→Waist	0.587	0.043	<0.001	0.531	0.027	<0.001
MSF 2→Log Glu	0.379	0.037	<0.001	0.529	0.027	<0.001
MSF 2→SBP	0.493	0.042	<0.001	0.635	0.025	<0.001
Covariance						
Periodontal disease↔MSF 1	0.096	0.041	0.020	0.083	0.038	0.027
Periodontal disease↔MSF 2	0.074	0.050	0.141	0.182	0.045	<0.001
Periodontal disease↔Teeth	−0.309	0.033	<0.001	−0.312	0.029	<0.001
Teeth↔MSF 1	−0.008	0.038	0.837	−0.019	0.031	0.548
Teeth↔MSF 2	−0.056	0.047	0.233	−0.079	0.039	0.045
MSF 1↔MSF 2	0.585	0.061	<0.001	0.473	0.047	<0.001
Direct effect						
Age→Periodontal disease	0.055	0.033	0.100	0.222	0.031	<0.001
Smoking→Periodontal disease	0.176	0.032	<0.001	0.068	0.031	0.027
Toothbrushing→Periodontal disease	−0.069	0.035	0.048	−0.076	0.031	0.014
Age→Teeth	−0.329	0.027	<0.001	−0.447	0.022	<0.001
Smoking→Teeth	−0.198	0.028	<0.001	−0.071	0.025	0.004
Toothbrushing→Teeth	−0.011	0.029	0.713	−0.012	0.025	0.617
Age→MSF 1	−0.245	0.039	<0.001	0.205	0.032	<0.001
Smoking→MSF 1	0.230	0.041	<0.001	0.132	0.031	<0.001
Alcohol→MSF 1	0.018	0.053	0.732	0.009	0.032	0.785
Medicine 1*→MSF 1	0.057	0.039	0.144	0.116	0.032	<0.001
Medicine 2†→MSF 1	0.103	0.045	0.022	0.091	0.034	0.007
Age→MSF 2	−0.018	0.050	0.725	0.340	0.035	<0.001
Smoking→MSF 2	0.121	0.043	0.004	−0.031	0.033	0.346
Alcohol→MSF 2	0.113	0.045	0.012	0.117	0.033	<0.001
Medicine 1→MSF 2	0.120	0.044	0.006	0.109	0.035	0.002
Medicine 2→MSF 2	0.383	0.045	<0.001	0.297	0.035	<0.001
Model fit index						
*χ*2	265.469			169.762		
df	47			47		
CFI	0.876			0.950		
RMSEA	0.067 (0.059–0.075)			0.044 (0.037–0.052)		
SRMR	0.037			0.030		

PD, pocket depth;%BOP, the percentage of teeth that bled on probing; Log TG, log triglycerides; Log Glu, log fasting glucose; SBP, systolic blood pressure; MSF, MS factor. *β*, standardized coefficient; SE, standard error.

* Anti-hyperlipidemic drug use.

† Anti-hypertensive drug or anti-diabetes drug use.

## Discussion

In this study, a gender difference in the association between periodontal disease and MS components was identified by logistic regression analysis (Table 2). There was a significant association between periodontal disease and MS in females, but not in males, depending on the cut-off value of mean PD defined as periodontal disease. Our results were similar to those of Andriankaja et al. (2010) that females having three or more MS components had a stronger association with periodontal disease than those with no MS component; however, this was not seen in males. In contrast, Kwon et al. (2011) reported no gender difference in the relationship between periodontal diseases and MS, although the subjects in their and our studies were both Mongolians. The result of logistic regression analysis is generally affected by disease definition. Subjects in the study by Kwon et al. (2011) were assessed using the WHO community periodontal index (CPI) and the definition of periodontal disease was a CPI greater than or equal to “code 3” at least one site among those assessed. In contrast, we used the NHANES III criteria for periodontal assessment. This difference in the definition of periodontal disease may explain the discrepancy between the report by Kwon et al. (2011) and the present one. In fact, in our study the association between periodontal disease and MS statistically differed in several definitions of periodontal disease. In males, the association between MS and periodontal disease defined by a mean PD ≥2.0 or 2.5 mm was statistically significant, but not for periodontal disease defined by a mean PD ≥3.0 or 3.5 mm. Thus, the different mean PD cut-off values may derive different statistical results in the analysis of association between periodontal disease and MS, and it is possible to have a lack of consistency of results due to different definition of periodontal disease. This indicates a defect of the usual approach and a need to evaluate periodontal disease qualitatively.

To overcome the difficulty mentioned above, we treated periodontal disease as a continuous variable. The causal and correlation relationships among variables concerned were analysed SEM. As a result, the MS single-factor model (Model 1) failed to provide a good fit to the observed data. To construct an alternative MS factor structure, factor analysis of MS components was carried out. Factor analysis has been used to reduce inter-related variables into a smaller set of factors. As the MS components were high inter-related, factor analysis resulted in two summary factors. The variables with a factor loading 0.40 were commonly used for interpretation of factors. We found that triglycerides and HDL loaded on one factor, that is, MSF1, and waist circumferences, fasting glucose, and systolic blood pressure on another factor, MSF2. In male, although fasting glucose had loading 0.37 on MSF2, because of close to 0.40, we considered that fasting glucose loaded on MSF2. The present factor analysis identified two summary factors, and this result suggest that one MS component alone (e.g. fasting glucose) does not underlie all features of MS and that more than one pathophysiological mechanism are present for the full expression of MS (Wang et al. 2004). Considering pathophysiologically, visceral obesity may be linked to decreased glucose tolerance with hyperinsulinemia, leading to reduced insulin-mediated glucose uptake (Timar et al. 2000). Indeed, obesity was associated with insulin resistance in previous studies (Edwards et al. 1994, Lempiainen et al. 1999). Insulin resistance with resulting compensatory hyperinsulinemia activates sympathetic activity that subsequently causes hypertension (Reaven et al. 1996). These interrelationships may explain why waist circumferences, fasting glucose and systolic blood pressure were related MSF2. On the other hand, in the pathophysiology of metabolic syndrome, increased triglyceride levels decrease the content of cholesterol HDL (Eckel et al. 2005); consequently, a cluster of triglycerides and HDL seems reasonable. As shown in factor analysis, it may be appropriate to assume two latent variables (i.e. factors) that explain the MS components in SEM.

According to the factor analysis, when an MS two-factor model was constructed for both genders, the model fitted female data well and but did not provide a good fit to the male data. This may suggest a gender difference in the association between periodontal disease and MS. There are several possible explanations for this gender difference. First, the different levels of MS inflammation in males and females may be related to differences in the association between periodontal disease and MS. Females with MS had higher levels of CRP than males (Saltevo et al. 2008), suggesting that the systemic inflammation level was higher in females than in males with MS. On the other hand, periodontal disease is associated with elevated CRP, but this has not been reported separately by gender (Slade et al. 2000, Noack et al. 2001). Because both periodontal disease and MS are associated with systemic inflammation, these health conditions are likely to be linked through a common pathophysiological pathway (D’Aiuto et al. 2008). The higher inflammation in females with MS could explain the stronger association of periodontal disease with MS in females than in males. In addition, our study showed that latent variable “Periodontal disease” was significantly related to “MSF2”, which was comprised of waist circumferences, fasting glucose and systolic blood pressure, in females, but not in males. Obesity is associated with systemic inflammation (Thorand et al. 2003) and periodontal disease (Saito et al. 2005). Because of higher inflammation in females with MS than in males, it is suggested that obesity-related factor is stronger associated with periodontal disease in females than in males. It is possible that males have a weak association between periodontal disease and MS; thus the male model did not fit the data well. Because the male model could not be explained using the observed variables, whether periodontal disease and MS are associated with factors or covariates not considered here should be investigated in future studies.

Another reason for the gender difference in the association between periodontal disease and MS may be the role played by sex hormones in the inflammatory process. Inflammatory cytokines decrease estradiol production by granulosa cells, suggesting that chronic inflammation may reduce the protective effect of oestrogen on body fat distribution and insulin action (Alpizar & Spicer 1994). Although oestrogen alone does not induce periodontal disease, it alters the periodontal tissue condition in response to the plaque microbiota (Mealey & Moritz 2003, Figuero et al. 2013).That is, systemic inflammation promoted by obesity could influence the production of oestrogen, which, in turn, affects insulin resistance and lipid metabolism and contributes to periodontal disease in females.

Considering the differences between males and females for biological factors as represented by sex hormones, gender difference in health status and disease condition is implied. As various aspects of gender differences have been recently studied (Klein et al. 2010, Eshima et al. 2011, 2012, Flanagan et al. 2011), it is important to pay attention to gender differences in the association between periodontal disease and MS. Understanding gender differences may lead to studying common preventive approach and treatment strategy for periodontal disease and MS, and gender differential approaches may be established. Further studies are expected to identify a more detailed relationship structure between periodontal disease and MS.

This study has several limitations. We did not consider other factors that may be related to periodontal disease and MS, such as socioeconomic status, inflammation indices (e.g. CRP) or psychosocial factors. To get into a more detail investigation of the mechanism of the gender difference in the association between periodontal disease and MS, these factors should be treated in future studies. In addition, there is a room for consideration of a method of assessment of periodontal disease in this study, NHANES III method. Since this method does not include examination of lingual or palatal sites susceptible to periodontal disease, it is possible that the prevalence of periodontal disease was underestimated. If the prevalence of periodontal disease affects the association between periodontal disease and MS, our findings may have to be reconsidered. To our knowledge, there is no report of such an effect. We conducted multiple statistical tests such as the logistic regression analysis, the factor analysis, and the SEM. This may cause multiplicity problems (Bender & Lange 2001), which is derived from the limitation of the present statistical techniques.

In conclusion, our data suggest gender differences in the association between periodontal disease and MS in both the quantitative and qualitative sense. This gender difference should be considered when the association between periodontal disease and MS is evaluated.

**Clinical Relevance**

*Scientific rationale for the study*: Gender differences in the prevalence of periodontal disease and metabolic syndrome have been reported. However, the existence of a gender difference in the association between the two health conditions has not yet been determined.

*Principal findings*: There was a gender difference in the association between periodontal disease and metabolic syndrome in both the quantitative and qualitative sense. The association in females was more consistent than that in males.

*Practical implications*: To precisely evaluate the association between periodontal disease and factors of metabolic syndrome, the gender difference must be considered.

## Conflict of interest and source of funding statement

The authors have no conflicts of interest to declare. This study was supported by Grants-in-Aid of Scientific Research (22390401, 22406034 and 23390483) from the Ministry of Education, Science, Sports, and Culture of Japan, Tokyo, Japan and by Health and Labour Sciences Research Grants from the Japanese Ministry of Health, Labour and Welfare.

## Supplementary material

Table S1 Pearson’s correlations between periodontal disease variables and the metabolic components examined.

Table S2 Periodontal disease odds ratios adjusted for drug treatment for elevated glucose and confounding factor.
