# Supplementary material for: Gender differences in the association between metabolic syndrome and periodontal disease: the Hisayama Study
Source: J Clin Periodontol. 2013 Jul 8;40(8):743–52. doi: 10.1111/jcpe.12119 (PMC3807558; doi:10.1111/jcpe.12119)
Supplement: Table S1 — Pearson’s correlations between periodontal disease variables and the metabolic components examined. [file jcpe0040-0743-sd1.doc]

| Supplementary Table 1. Pearson’s correlations between periodontal disease variables and the metabolic components examined. | | | | | | | | | | | |  |  |
| --- | --- | --- | --- | --- | --- | --- | --- | --- | --- | --- | --- | --- | --- |
| Variable | Males | | | | | |  | Females | | | | | |
|  | PD | %BOP | Log TG | HDL | SBP | Waist |  | PD | %BOP | Log TG | HDL | SBP | Waist |
| %BOP | 0.63^***^ |  |  |  |  |  |  | 0.60^***^ |  |  |  |  |  |
| Log triglycerides (TG) | 0.09^**^ | 0.03 |  |  |  |  |  | 0.10^**^ | 0.10^***^ |  |  |  |  |
| High density lipoprotein (HDL) | -0.10^**^ | -0.04 | -0.46^***^ |  |  |  |  | -0.13^***^ | -0.11^***^ | -0.50^***^ |  |  |  |
| Systolic blood pressure (SBP) | 0.08^**^ | 0.08^**^ | 0.21^***^ | 0.01 |  |  |  | 0.13^***^ | 0.16^***^ | 0.28^***^ | -0.15^***^ |  |  |
| Waist circumference | 0.06 | 0.01 | 0.27^***^ | -0.34^***^ | 0.28^***^ |  |  | 0.14^***^ | 0.08^**^ | 0.28^***^ | -0.25^***^ | 0.34^***^ |  |
| Log fasting glucose | 0.02 | 0.01 | 0.13^***^ | -0.08^*^ | 0.23^***^ | 0.22^***^ |  | 0.13^***^ | 0.09^**^ | 0.24^***^ | -0.16^***^ | 0.33^***^ | 0.29^***^ |
| ^*^ *p* < 0.05, ^**^ *p* < 0.01, ^***^ *p* < 0.001 | |  |  |  |  |  |  |  |  |  |  |  |  |
