# Supplementary material for: Gender differences in the association between metabolic syndrome and periodontal disease: the Hisayama Study
Source: J Clin Periodontol. 2013 Jul 8;40(8):743–52. doi: 10.1111/jcpe.12119 (PMC3807558; doi:10.1111/jcpe.12119)
Supplement: Table S2 — Periodontal disease odds ratios adjusted for drug treatment for elevated glucose and confounding factor. [file jcpe0040-0743-sd2.doc]

| Supplementary Table S2. Periodontal disease odds ratios adjusted for drug treatment for elevated glucose and confounding factor | | | | | | | | | |
| --- | --- | --- | --- | --- | --- | --- | --- | --- | --- |
| Accumulation of metabolic components | | | Males | |  | Females | |  | Interaction for  gender × metabolic component  *p*-value^‡^ |
|  |  |  | Adjusted OR^†^ (95% CI) | |  | Adjusted OR^†^ (95% CI) | |  |  |
| **Mean PD ≥ 2.0 mm** | | |  |  |  |  |  |  |  |
|  | Number of metabolic components | |  |  |  |  |  |  |  |
|  |  | 0 component | 1 |  |  | 1 |  |  |  |
|  |  | 1 and 2 component | 1.15 | (0.75–1.78) |  | 1.28 | (0.92–1.78) |  | 0.528 |
|  |  | ≥ 3 components (metabolic syndrome) | 1.54 | (0.95–2.49) |  | 1.50 | (1.03–2.18) |  | 0.779 |
| **Mean PD ≥ 2.5 mm** | | |  |  |  |  |  |  |  |
|  | Number of metabolic components | |  |  |  |  |  |  |  |
|  |  | 0 component | 1 |  |  | 1 |  |  |  |
|  |  | 1 and 2 component | 1.37 | (0.87–2.15) |  | 1.46 | (0.94–2.28) |  | 0.655 |
|  |  | ≥ 3 components (metabolic syndrome) | 1.73 | (1.08–2.77) |  | 1.69 | (1.05–2.73) |  | 0.622 |
| **Mean PD ≥ 3.0 mm** | | |  |  |  |  |  |  |  |
|  | Number of metabolic components | |  |  |  |  |  |  |  |
|  |  | 0 component | 1 |  |  | 1 |  |  |  |
|  |  | 1 and 2 component | 1.30 | (0.75–2.25) |  | 2.04 | (0.97–4.30) |  | 0.295 |
|  |  | ≥ 3 components (metabolic syndrome) | 1.21 | (0.68–2.14) |  | 2.93 | (1.35–6.35) |  | 0.035 |
| **Mean PD ≥ 3.5 mm** | | |  |  |  |  |  |  |  |
|  | Number of metabolic components | |  |  |  |  |  |  |  |
|  |  | 0 component | 1 |  |  | 1 |  |  |  |
|  |  | 1 and 2 component | 0.99 | (0.49–1.99) |  | 2.62 | (0.77–8.96) |  | 0.134 |
|  |  | ≥ 3 components (metabolic syndrome) | 1.11 | (0.54–2.28) |  | 3.59 | (1.02–12.63) |  | 0.067 |
| Logistic regression analysis with periodontal disease (mean PD cut-off value ≥ 2.0, 2.5, 3.0, 3.5, or 4.0 mm) as the dependent variable and accumulation of metabolic components as the independent variable. | | | | | | | | | |
| ^†^Adjusted for drug treatment for elevated glucose, age, smoking habits, alcohol intake, toothbrushing frequency, and present number of teeth. | | | | | | | | | |
| ‡*p*-value of the interaction term consisting of gender and MS components were calculated by using gender, MS components, drug treatment for elevated glucose, age, smoking habits, alcohol intake, toothbrushing frequency, and present number of teeth as explanatory variables. The interaction term was created by multiplying gender variable (0 = male, 1 = female) by MS components variable (0 = 0 component, 1 = 1 and 2 component, 2 = 3 or more components). | | | | | | | | | |
